# From the lab to the real world: emotions serving morality in dyadic negotiation

**DOI:** 10.3389/fnbeh.2026.1764703

**Published:** 2026-02-25

**Authors:** Michela Balconi, Roberta A. Allegretta, Angelica Daffinà

**Affiliations:** 1 International Research Center for Cognitive Applied Neuroscience (IrcCAN), Università Cattolica del Sacro Cuore, Milan, Italy; 2 Research Unit in Affective and Social Neuroscience, Department of Psychology, Università Cattolica del Sacro Cuore, Milan, Italy

**Keywords:** brain-to-brain, cognitive system, emotional system, fNIRS, hyperscanning, moral negotiation

## Abstract

**Introduction:**

Confronting moral choices in contexts of limited resources requires individuals to integrate reasoning, emotions, and interpersonal dynamics. However, most research on moral decision-making relies on laboratory paradigms that limit ecological validity, restricting natural emotional expression. To address this limitation, this study examined how dyads converge on moral choices through real-time negotiation, focusing on the interplay between cognitive and emotional processing.

**Methods:**

Fifteen same-sex adult dyads participated in a moral evaluation task, deciding which of two patients to prioritise for treatment. During the negotiation, conducted in direct social interaction rather than in isolated lab-based evaluation, prefrontal cortex activity was simultaneously recorded in both participants using fNIRS hyperscanning, a paradigm suited to naturalistic interpersonal contexts.

**Results:**

Results revealed a significant increase in the dissimilarity in the deoxygenated haemoglobin (HHb) activity between channel 6 (F6-F4, right hemisphere) and channel 3 (F5-F3, left hemisphere);no significant effects were observed for oxygenated haemoglobin (O_2_Hb); This, may suggest a differentiated engagement of analytical reasoning (left hemisphere) and emotional–social processing (right hemisphere). The latter – expressed through subtle embodied cues—plays a central regulatory role in influencing each other’s judgment.

**Discussion:**

These findings support the view that moral negotiation is a dynamic, affectively grounded process, shaped not only by cognitive deliberation but also by emotional information expressed through bodily and facial cues. By integrating hyperscanning with a naturalistic interpersonal setting, this study can contribute to bridging the gap between lab-based and real-world moral decision-making, offering insights into the neural underpinnings of shared evaluation.

## 1 Introduction

In professional or everyday settings, individuals frequently face moral choices that impact others’ well-being, such as prioritizing patients in an overcrowded emergency room or allocating scarce resources.

These decisions, especially in critical and emergency contexts, are inevitable and complex, requiring evaluations under uncertainty and emotional pressure. They involve balancing benefits and ethical conflicts (e.g., sacrificing one for many), relying not only on objective evaluations but also on applying fundamental moral principles like distributive justice, fairness, merit, and utilitarianism. These principles are influenced by the external context and emotional involvement (O’Fallon and Butterfield, 2005; Kish-Gephart et al., 2010). In this sense, moral judgment can be defined as the process of evaluating the ethical acceptability of a behavior or a series of behaviors (Reidenbach and Robin, 1988). Recent affective neuroscience research highlights that these choices unfold within dynamic social interactions, where non-verbal emotional cues (such as facial expressions, gaze, body posture, and vocal tone) act as communicative channels that convey empathy, agreement, or tension (Kret and de Gelder, 2012; de Gelder, 2009). Recognizing these embodied components is important for achieving ecological validity when studying moral behavior.

Neuroscience and cognitive psychology have adopted experimental methodologies aimed at analysing the mechanisms underlying moral judgment. Among these, the use of hypothetical moral dilemmas has proven particularly useful, as they allow for isolating and examining the cognitive and emotional processes involved in moral decision-making, in the absence of real-world consequences (Sinnott-Armstrong, 1987; Tasso et al., 2017; Palmiotti et al., 2020).

Moral judgment is thought to arise from a conflict between utilitarian and deontological perspectives: the former maximising collective well-being even when this is achieved through inequality, the latter adhering to moral norms regardless of outcomes (Greene et al., 2004, 2008). According to the dual-process model of moral cognition, utilitarian choices depend on cognitive control, whereas deontological ones are emotionally driven (Greene et al., 2001; Haidt, 2001). Thus, within this framework, emotions cannot be considered peripheral events but play a central role, acting as communicative and motivational forces, dynamically influencing attention, empathy, and judgment through expressive and physiological dimensions.

To further explore the neural substrates underlying these cognitive and emotional mechanisms, neuroimaging studies have examined which brain regions are engaged during moral decision-making, such as the dorsolateral prefrontal cortex (DLPFC), anterior cingulate cortex (ACC), and anterior insula during moral decision-making; with the ACC particularly involved in emotionally complex dilemmas (Greene, 2007; Hutcherson et al., 2015).

However, these are limited in capturing the dynamic and reciprocal nature of real-time social interactions.

To address this limitation and to investigate the neurocognitive dynamics underlying real-time interpersonal interactions involving joint moral evaluation, recent research employs the use of hyperscanning to record simultaneous brain activity from multiple participants. In particular, functional near-infrared spectroscopy (fNIRS) has been largely used because it allows the analysis of hemodynamic signals resulting from prefrontal cortex (PFC) activation, being comparable with BOLD signals obtained by fMRI (Sato et al., 2013); allowing for the explanation of neuronal activation in terms of an increase in oxygenated haemoglobin (O_2_Hb) and a concomitant decrease in deoxygenated haemoglobin (HHb). This is indicative of an increase in local metabolic activity. Conversely, the opposite pattern has been interpreted as indicative of neural inhibition or reduced activation in specific cortical areas. In particular, the HHb component has been linked to inhibitory processes, as its increase is frequently regarded as a potential indicator of functional suppression, especially during the execution of cognitively demanding tasks (Cui et al., 2010; Hoshi, 2016; Balconi and Vanutelli, 2017a; Acconito et al., 2025).

More specifically, Dashtestani et al. (2018) employed fNIRS to investigate the brain’s hemodynamic activity during moral decision-making, finding that impersonal (cognitive) moral dilemmas elicited greater O_2_Hb changes in PFC, particularly in the left DLPFC, compared to personal (emotional) dilemmas. This effect was especially noticeable during non-utilitarian responses, suggesting a critical interplay between emotional and rational processing in moral judgment and validating fNIRS utility for exploring hemodynamic correlates in individual contexts. Recognizing that moral decisions are often social, the integration of fNIRS during hyperscanning offers a promising development for future research to study real-time, shared moral evaluation (Balconi and Vanutelli, 2017a; Quaresima and Ferrari, 2019; Acconito et al., 2025).

To address the lack of research on joint ethical evaluation, the concept of negotiated moral decision-making was introduced. This process requires two or more individuals to confront each other to reach an agreement on a morally relevant choice. It entails integrating diverse perspectives, balancing personal versus collective beliefs, and adopting an ethically defensible solution, and the need for advanced skills such as empathy, theory of mind, emotional regulation, and acceptance of compromise (Decety and Cowell, 2014). Its complexity stems from the fact that participants must confront deeply personal values often perceived as non-negotiable, which resonate with their own moral identity. Also, reciprocal affective responses can play a pivotal role, influencing moral evaluation, also through the manifestation of subtle interpersonal cues, such as facial expression, voice tone, gaze, and body posture. These spontaneous signals, in fact, could reflect how emotions are conveyed, perceived, and managed during shared moral evaluation. Thus, these dynamics have the potential to induce cognitive and emotional tensions, which can limit the establishment of a shared agreement, particularly in the absence of explicit normative references or collectively recognized objective criteria.

The investigation of negotiated moral decisions can be facilitated by the utilization of structured dyadic dilemmas, such as those concerning the allocation of life-saving care under conditions of limited resources (Persad et al., 2009; Balconi and Fronda, 2020). In such contexts, decisions must be made collectively, despite the absence of a clearly defined normative hierarchy. This process involves the deliberate exclusion of at least one ethically valid option and necessitates the reassessment of one’s moral positions, frequently influenced by anticipatory emotions such as guilt and fear of negative relational consequences (Gangemi et al., 2025).

In the present study, participants, organized in pairs, are asked to imagine themselves in the position of a physician forced to choose which of two patients to prioritize in treatment in a hypothetical pandemic emergency scenario. They negotiate a shared, ethically defensible solution, enabling the exploration of decision-making and neural dynamics of moral negotiation. Also, this approach thus allows the exploration of interbrain dynamics underlying affective attunement that often manifests through non-verbal cues. Recent literature distinguishes between social, affective, and informative gestures, each fulfilling distinct communicative functions such as initiating, maintaining, or interrupting interaction, expressing emotional states, or directing attention (Bavelas et al., 1992; Tomasello et al., 2005; Enfield et al., 2007; Kendon, 2017), contributing to the perception of inclusion, empathy, and cooperation (Balconi and Vanutelli, 2017b).

In line with the specific features of the proposed task, it is hypothesized that when participants are not aligned, either emotionally or cognitively, higher dissimilarity in the relevant brain areas will be observed, consistent with their neurofunctional properties. These patterns, modulated by the nature of the misalignment between partners, could represent dynamic indicators of the difficulty of achieving a shared moral agreement.

Specifically, cognitive misalignment between dyad members, such as differences in reasoning processes, normative interpretation, or analytical evaluation of the proposed situation, is expected to result in increased hemodynamic dissimilarity in the left prefrontal areas, which are traditionally associated with cognitive control and deliberative thinking. Conversely, when misalignment is emotional or affective (for example, due to differences in the intensity or quality of empathic responses), an increase in dissimilarity is expected in the activity of the right prefrontal areas, which are associated with emotional regulation and social cognition.

## 2 Materials and methods

### 2.1 Sample

The study sample included 30 young adults (18 women, mean age = 23.44 years, SD = 1.92; 12 males, mean age = 23.75, SD = 1.92), recruited via convenience sampling without probabilistic criteria. Participants were randomly assigned and grouped into 15 same-sex pairs (9 female, 6 male). To avoid relational bias and ensure the independence of interpersonal dynamics, any prior acquaintance between dyad members was strictly excluded. Participation was voluntary and unpaid.

Participants were required to have normal or corrected-to-normal vision and no history of psychiatric or neurological disorders. Those with significant depressive symptoms, cognitive impairments, or under psychoactive treatment would have been excluded based on clinical history and self-report at screening. No participant met these exclusion criteria, and all recruited participants were included in the study. All procedures complied with international ethical standards and were approved by the Ethics Committee of the Department of Psychology at Università Cattolica del Sacro Cuore, Milan, in accordance with the Declaration of Helsinki (2013) and the European GDPR (EU Regulation 2016/679).

### 2.2 Procedure

**Figure 1:**
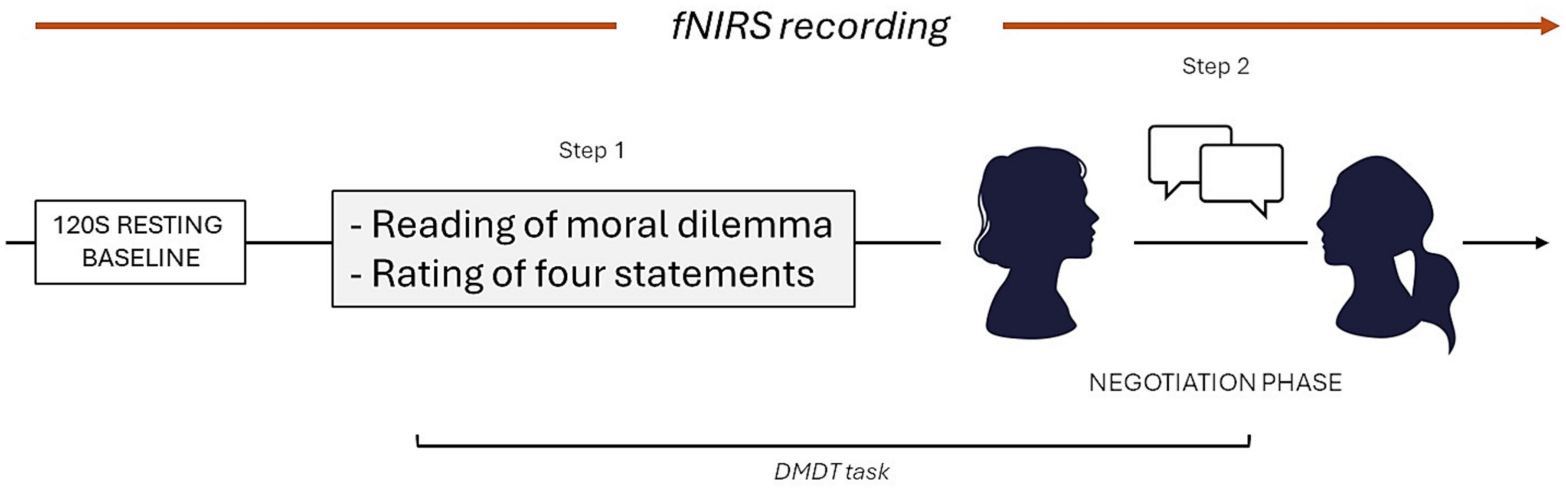
Experimental procedure: Experimental setup for the Dyadic Moral Evaluation Task (DMET). Participants sat facing each other and were simultaneously recorded using fNIRS hyperscanning while completing a two-part task: (1) An individual evaluation to determine their primary reasoning style (PRS—emotional vs. cognitive) in response to a morally challenging medical scenario, and (2) a joint discussion aimed at reaching a mutual decision.

Before initiating the experimental protocol, all participants received standardized written and verbal instructions to ensure clear understanding and consistent adherence to procedural guidelines. The experimental session took place in a sound-attenuated room and lasted in total approximately 30 min (Figure 1). Throughout the session, participants were instructed to maintain a stable posture, articulate their responses clearly, and alternate speaking turns to avoid overlapping speech. Audio-video recordings of the full session were collected to monitor and annotate significant non-verbal behaviors (e.g., facial expressions, posture changes) that could influence interpersonal dynamics. These recordings also provided potential behavioral indicators of emotional engagement, offering a possible external complement to the neurophysiological data.

Participants were seated face-to-face to enable direct verbal communication while minimizing external distractions. fNIRS data were recorded simultaneously from both members of each dyad using a hyperscanning setup, during two separate phases: a 120 s resting-state baseline and the Dyadic Moral Evaluation Task (DMET) phase, during which participants engaged in an interactive moral reasoning task.

The DMET consisted of two sequential steps; during Step 1, participants were presented with a morally charged scenario, stating that in a hospital overwhelmed by the SARS-CoV-2 outbreak, a physician faced a morally challenging situation: deciding which of two elderly patients, both in their eighties, should be given priority for treatment. One patient was described as a widower with hypertension who had received long-term care from his chronically ill daughter, who was said to be anxiously awaiting news of his condition. The other patient was reported to be diabetic. These distinctions were designed to elicit emotional engagement while maintaining medical equivalence.

Participants were asked to consider the decision-making scenario and indicate which patient they would prioritize for treatment if acting as the doctor. They then rated their agreement with four justifications for patient prioritization on a five-point Likert scale (1 = complete disagreement; 5 = complete agreement).

The statements were designed to reflect two implicit evaluative approaches—emotional and cognitive—without explicitly naming them to prevent bias. Two items emphasized emotional factors such as empathy and relational concern (e.g., the patient’s dual vulnerability due to illness and caregiving responsibilities, or the severity and suffering linked to his condition), while the other two adopted a more cognitive focus, emphasizing diagnostic and prognostic considerations.

Instead, regarding the other two cognitive justifications, one pointed out the better short-term prognosis of the second patient, arguing that diabetes implies less immediate danger than severe hypertension and thus presented a greater chance of successful treatment. The final argument emphasized operational efficiency, proposing that the diabetic patient, if stabilized quickly, could be discharged sooner, allowing medical resources to be allocated to more critical cases.

From these responses, two continuous scores were computed: (i) Emotional Oriented Score (EOS): average agreement with the emotionally framed justifications; (ii) Cognitive Oriented Score (COS): average agreement with the cognitive framed justifications. Afterwards, these two scores were compared to determine each person’s Primary Reasoning Style (PRS): PRS-Emotional (PRS-E; 10 participants) if the participant agreed more with the emotional justifications; PRS-Cognitive (PRS-C; 13 participants) if the participant agreed more with the rational justifications, and PRS-Balanced (PRS-B; 6 participants) if the scores were equal (up to the second decimal point). One participant was excluded because at least one of his answers was not correctly recorded. This individual classification enabled the creation of a higher-level dyadic variable, the Dyadic Reasoning Index (DRI), indicating the level of moral reasoning alignment within each of the remaining 14 pairs. Dyads with matching reasoning styles (both PRS-E, both PRS-C, or both PRS-B) were labelled DRI-Aligned (DRI-A; 4 dyads), while those with mixed styles were categorized as DRI-Misaligned (DRI-M; 10 dyads). This treatment of PRS-B was chosen in relation to the exploratory characteristics of the study, given the small sample of dyads.

After completing the individual assessment, during Step 2, dyads progressed to a joint deliberation phase. Participants were instructed to negotiate and agree unanimously on which patient to prioritize, selecting one of the four previously evaluated justifications. This negotiation was limited to a maximum of 180 s, introducing time constraints intended to simulate real-world decision pressure and avoid excessive cognitive deliberation. This two-phase structure (individual assessment followed by joint negotiation) was designed to closely approximate real-world decision-making dynamics in critical contexts, where individuals must first form a personal judgment and then collaboratively reach a shared solution under time pressure, even if it remains hypothetical.

### 2.3 fNIRS data acquisition and processing

Using a six-channel optodes array from the NIRScout System (NIRx Medical Technologies, LLC, Los Angeles, CA, USA), changes in oxygenated haemoglobin (O_2_Hb) and deoxygenated haemoglobin (HHb) concentrations were recorded. Four light sources and four detectors were arranged on the scalp through an fNIRS cap, following the international 10/5 placement system (Oostenveld et al., 2001). The distance between each emitter and detector pair was set at 30 mm, using two near-infrared wavelengths (760 nm and 850 nm). The placement of optodes and their spacing was guided by a probabilistic brain atlas available within the fOLD software (fNIRS Optodes’ Location Decider, version 2.2.1) (Zimeo Morais et al., 2018), which ensured correct alignment with relevant functional brain areas and Brodmann regions (Koessler et al., 2009; Giacometti et al., 2014). The focus was on the frontal cortex, specifically the PFC, to assess its involvement during the evaluation task. Signals were collected at a sampling frequency of 6.25 Hz using NIRStar Acquisition Software version 12.4 (NIRx Medical Technologies LLC, Glen Head, NY, USA). Data were then processed and transformed with nirsLAB software (v2014.05; NIRx Medical Technologies LLC), resulting in mmol·mm values that represent O_2_Hb and HHb concentration changes per channel. To improve data quality during preprocessing, a digital band-pass filter ranging from 0.01 to 0.3 Hz was applied to the raw haemoglobin signals (Balconi and Vanutelli, 2017b; Balconi et al., 2019).

**Figure 2:**
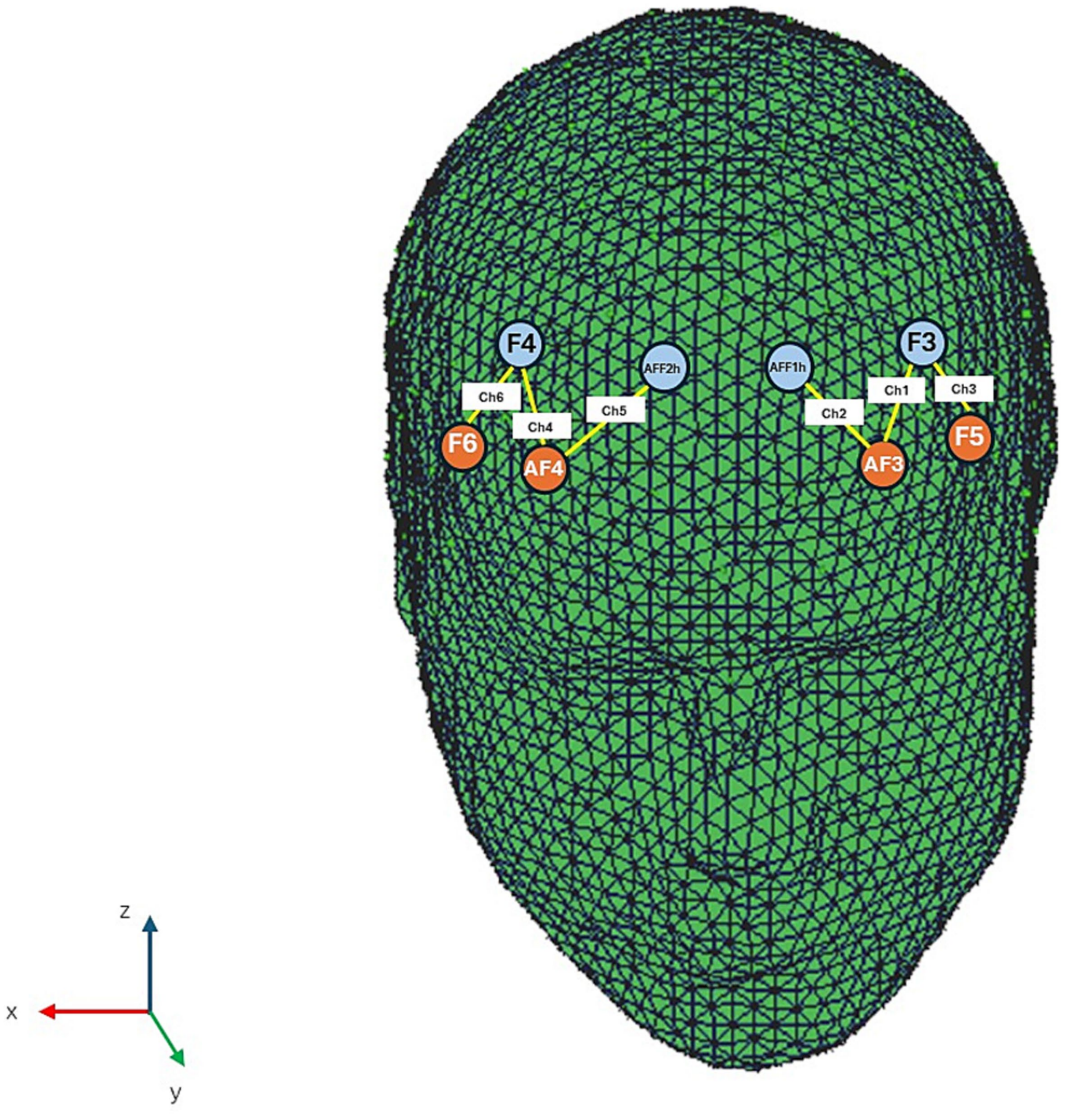
Head rendering showing the fNIRS montage configuration, with emitters (AF3, AF4, F5, and F6) marked in orange and detectors (AFF1h, AFF2h, F3, and F4) in light blue. The six active measurement channels are shown in yellow: Ch1 (AF3 to F3), Ch2 (AF3 to AFF1h), Ch3 (F5 to F3), Ch4 (AF4 to F4), Ch5 (AF4 to AFF2h), and Ch6 (F6 to F4).

The raw time-series data were carefully examined visually for each participant throughout the experiment and during signal processing to identify channels affected by noise from movement artifacts or amplitude instability. Channels exhibiting poor optical contact or missing the ~1 Hz heartbeat rhythm were excluded from further analysis (Pinti et al., 2015). Additionally, a linear-phase Finite Impulse Response (FIR) filter with a cutoff at 0.3 Hz was employed to remove respiration-related signals, ensuring symmetrical filtering (Naseer and Hong, 2013; Naseer et al., 2014). After preprocessing, the average concentration of haemoglobin changes per channel across the different phases was calculated. The effect size for each experimental condition was quantified by measuring the difference in mean concentration between baseline and task periods for every channel and participant. Effect sizes (Cohen’s d) were computed as: D = (m1 − m2)/s, where m2 and m1 represent mean concentrations for baseline and task, respectively, and s denotes the baseline standard deviation. These effect sizes from the six channels were averaged to improve the signal-to-noise ratio. Although the raw fNIRS measurements are relative, averaging normalized effect sizes is valid since they are independent of the differential pathlength factor (DPF). Using this optode configuration, six channels were recorded: Ch1 (AF3-F3), Ch2 (AF3-AFF1h), and Ch3 (F5-F3) for the left prefrontal cortex (PFC), and Ch4 (AF4-F4), Ch5 (AF4-AFF2h), and Ch6 (F6-F4) for the right PFC (Figure 2).

### 2.4 Data analysis

To investigate inter-brain neural coupling between individuals, fNIRS data were assessed for each channel at the dyad level using Euclidean distance (EuDist) as a measure of dissimilarity, quantifying the extent to which, within each dyad, brain activity differs among individuals. EuDist provides a continuous, non-directional index of functional divergence: higher values indicate greater differences in activation patterns between members of the dyad, whereas lower values reflect lower differences; it does not aim to capture moment-to-moment temporal synchrony between participants, but rather to quantify convergence versus divergence of average functional activation states during the negotiation. This approach follows established protocols for analysing neural coupling between individuals (Angioletti et al., 2025; Balconi et al., 2025).

Average speech durations were calculated individually for each participant, including only negotiation-relevant speaking turns. Utterances unrelated to the evaluation task, as well as pauses, were excluded from the analysis. The mean speech durations and their standard deviations were then analysed to assess consistency between dyad members, ensuring that both participants engaged in the interaction with comparable timing dynamics.

Initially, a repeated-measures ANOVA was conducted to explore potential effects of dyadic reasoning similarity. The Dyadic Reasoning Index (DRI; 2 levels: DRI-Aligned, DRI-Misaligned) was included as a between-subject factor, while Channel (6 levels: Ch1 to Ch6) was treated as a within-subject factor. The dependent variable in each analysis was the dissimilarity index calculated for O_2_Hb and HHb, respectively. However, no significant main effects or interactions were observed for DRI or Channel in this analysis (all *p* > 0.05).

Therefore, since DRI was not significant in the first set of analysis, to achieve a more parsimonious statistical model with fewer comparisons, the non-significant factor was excluded from the ANOVA, and a subsequent repeated-measures ANOVA was conducted with Channel as the only within-subject factor, using the same dependent variables (O_2_Hb and HHb). Following the exclusion of the non-significant DRI factor, this Channel-only analysis allowed an exploratory examination of general inter-hemispheric divergence patterns across channels at the dyad level.

All analyses were conducted with Greenhouse–Geisser corrections when sphericity was violated, to appropriately adjust the degrees of freedom. Significant interaction effects were examined in more detail using pairwise comparisons, and Bonferroni corrections were applied to mitigate the risk of Type I errors associated with multiple testing. For significant findings, effect sizes were reported using eta squared (η^2^), and statistical significance was determined at an alpha level of 0.05. Prior to the main analyses, data normality was verified through assessments of skewness and kurtosis.

## 3 Results

**Figure 3:**
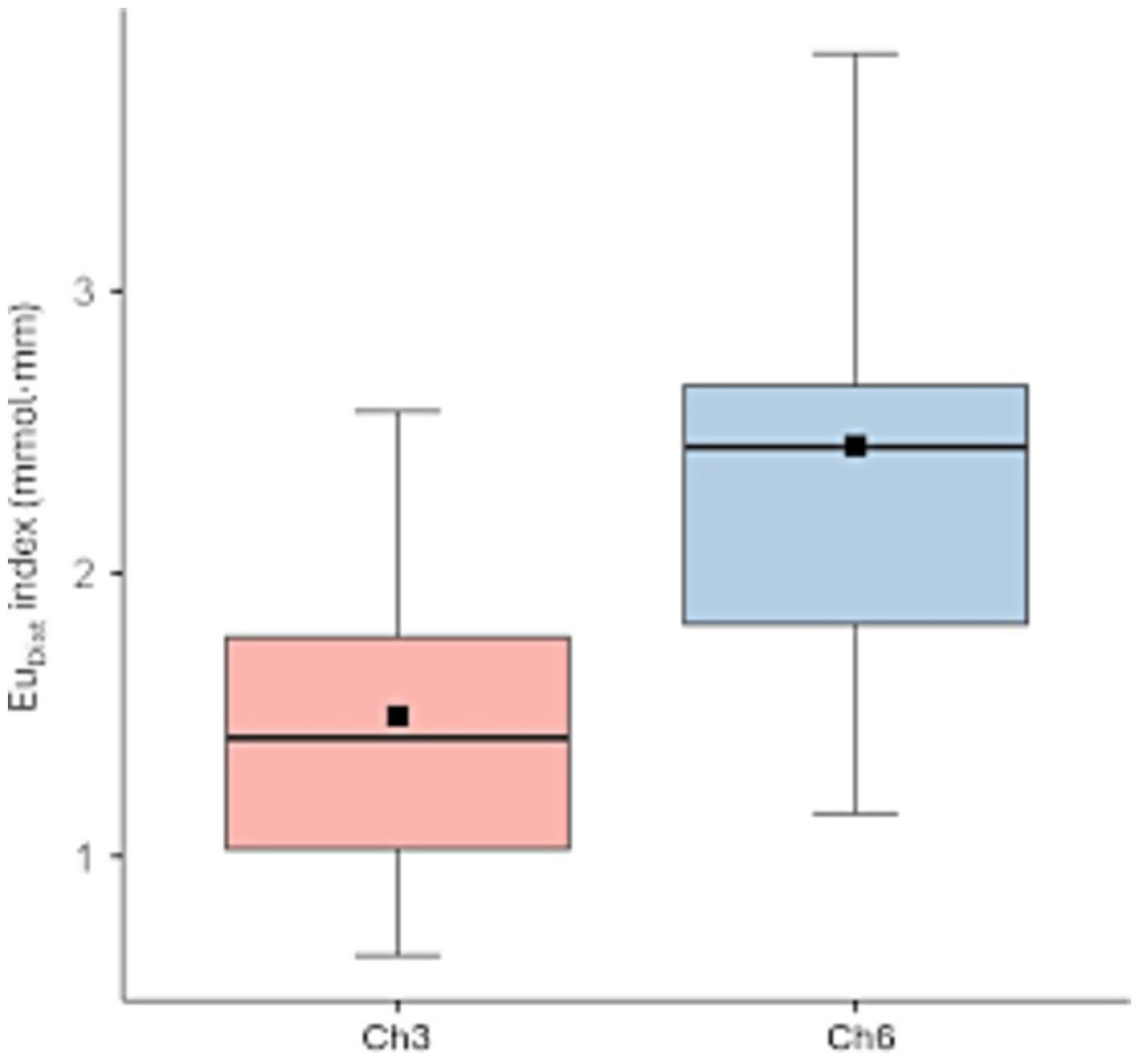
Boxplot illustrating the distribution of normalized Euclidean distance (EuDist, mmol·mm) values in the HHb between channel 6 and channel 3. The plots display interquartile range (boxed area) and median (central line). Statistical analyses revealed a significant main effect of channel, with *post hoc* Bonferroni-corrected comparisons indicating that EuDist was significantly higher in channel 6 compared to channel 3.

For HHb, the analysis with Channel only revealed a significant main effect [*F*_(3.21,45)_ = 3.20, *p* = 0.029, η^2^p = 0.186]. *Post hoc* comparisons (Bonferroni-corrected) revealed that EuDist (dissimilarity) was significantly higher for Channel 6 compared to Channel 3 (*p* = 0.022) (Figure 3).

No other significant results were found. No significant results were found for O_2_Hb (all *p* > 0.50).

As regards behavioral data, all but one of the pairs reached a final agreement by selecting at least one of the options initially suggested by the individual members. Only one pair found a “third way” to reach an agreement. On average, it took 2.30 min to reach an agreement. In terms of the content of the agreements, seven of the fourteen pairs opted for emotionally oriented justifications, while the remaining seven chose cognitively oriented justifications.

## 4 Discussion

The present study provides novel insights into the neurocognitive dynamics underlying negotiated moral decision-making in a realistic scenario inspired by the SARS-CoV-2 pandemic. Participants were presented with a realistic moral dilemma, requiring them to decide which of two elderly patients to prioritize in the event of resource constraints. This scenario gave rise to a conflict between competing moral principles, requiring integration of individual moral judgment with interpersonal coordination, approximating real-world shared decision-making (Decety and Cowell, 2014; Gangemi et al., 2025).

Although the DRI did not yield significant differences between aligned and misaligned dyads, this non-significant result is conceptually informative and suggests that neural dissimilarity occurs independently of the dyad’s reasoning alignment. Rather than being driven by specific dyadic reasoning styles, the observed pattern appears to reflect a more general feature of joint moral negotiation. In other words, the inter-hemispheric dissimilarity could reflect a general pattern of cognitive-emotional processing during joint moral negotiation, rather than being driven by specific dyadic styles.

On the other hand, the primary significant result shows a significant increase in dissimilarity in hemodynamic HHb activity between channel 6 (F6-F4, right hemisphere) and channel 3 (F5-F3, left hemisphere). In contrast, no significant differences emerged for O_2_Hb.

This inter-hemispheric dissimilarity likely reflects complementary engagement of analytical reasoning (left PFC) and socio-emotional processing (right PFC) (Greene et al., 2004; Killgore and Yurgelun-Todd, 2007; Decety and Cowell, 2014; Patil et al., 2021). Conflict between these systems may reflect the effort to reconcile rational evaluations (e.g., establishing a clinical priority) with empathic concern (e.g., significant family ties), a dynamic intensified by interpersonal interaction (Dashtestani et al., 2018; Balconi et al., 2025).

The lack of clear normative criteria likely accentuated the tension between rapid, intuitive emotional responses and controlled, deliberate reasoning (Greene et al., 2001; Haidt, 2001), possibly leading to differences in neural activation patterns(Cui et al., 2012). The hemispheric asymmetry may further index a functional conflict between utilitarian logic and deontological norms, as participants justified their own stance while negotiating with others (Treviño et al., 2006; Balconi and Fronda, 2020). From an affective neuroscience perspective, such hemispheric differentiation may be consistent with differential involvement of prefrontal regions in processes related to the regulation of emotional contagion and empathic resonance, whereby the right PFC has been suggested to play a role in modulating the perception and expression of others’ emotions during interactive moral evaluation. However, even if these processes may involve emotional regulation and empathic engagement, the results of this exploratory study do not allow for precise attribution to specific regulatory mechanisms.

Finally, the role of the proposed scenario should be considered: the emotionally charged pandemic framing likely intensified affective engagement, consistent with evidence that framing influences moral judgment and bias under uncertainty (Tversky and Kahneman, 1981; Navarick and Moreno, 2022; Gangemi et al., 2025).

Furthermore, the dissociation between the HHb and the O_2_Hb can be explained by their physiological differences. Generally, an increase in neuronal activity is associated with an increase in O_2_Hb as a result of increased oxygenated blood flow, accompanied by a decrease in HHb due to increased oxygen extraction by tissues. However, these signals are not always symmetrical and can be differentially influenced by systemic factors and artefacts. Indeed, the HHb component is often considered to be relatively more stable and less susceptible to physiological interference or motion artefacts, especially in complex protocols such as hyperscanning. This makes it easier to detect functional differences between the brain regions involved and may reflect local metabolic changes associated with neuronal activity more directly than O_2_Hb (Sato et al., 2013).

Taken together, these neurophysiological findings can be better understood if considering the embodied and interactive context in which moral negotiation occurs. In fact, although the present study aims to investigate the neurophysiological correlates of shared moral decision-making by examining the cognitive and emotional components of dynamic interaction rather than overt behavioral expressivity, acknowledging embodied dynamics provides an important conceptual background for interpreting interpersonal neural synchronization in realistic social contexts. The theoretical perspective offered by gesture and embodiment studies offers a valuable interpretive lens for understanding the interpersonal dynamics captured through hyperscanning, as the dialogic nature of the task likely involved the mutual perception and interpretation of subtle emotional cues (such as facial micro-expressions, tone of voice, and posture) that regulate interpersonal contact, convey affective information, and guide attention (Tomasello et al., 2005). In moral negotiation, such implicit expressive signals may represent the behavioral counterpart of the observed neural synchronization, accompanying verbal reasoning and emotional exchange while modulating empathy, inclusion, and cooperation (Balconi and Vanutelli, 2017b). Recognizing their potential involvement enhances the ecological validity of the neural findings and supports a socially grounded understanding of moral decision-making as a socio-emotional process.

Despite promising results, this study should be considered as preliminary due to the small sample size, and some limitations should be noted. Firstly, beyond visual inspection and exclusion of noisy channels, no automated motion correction algorithms were applied, and the optode configuration did not include short-separation channels. Although motion artefacts were minimized at the experimental level through task design and participant instructions, residual non-neuronal contributions to both O_2_Hb and HHb signals cannot be entirely excluded. Thus, the observed effects should be interpreted as relative patterns of inter-hemispheric divergence, rather than as direct indicators of specific neural mechanisms. Secondly, despite the present task incorporating a realistic moral dilemma and facilitating face-to-face negotiation, it remains a hypothetical scenario with a limited duration (180 s). Consequently, while the design is more interactive and naturalistic than standard isolated paradigms, such findings should not be generalized to real-world clinical decision-making. Thirdly, the complexity of the evaluation task makes it challenging to accurately identify the specific factors contributing to neural synchronization or dissimilarity, such as empathy, emotional engagement, and communication strategies. Future studies could integrate behavioral measures (e.g., detailed coding of negotiation strategies, turn-taking, and non-verbal cues)individual profiles (e.g., empathy and moral traits), and dialogical content analysis, including non-verbal cues, to better understand sources of disagreement and compromise strategies. Additionally, another research perspective could be to integrate tools for detecting autonomic indices, such as skin conductance or heart rate variability, to capture physiological components related to motivational activation and stress. Combining cortical neurophysiological data with peripheral autonomic signals would allow distinctions to be drawn between cognitive and emotional processes, thereby contributing to a deeper understanding of the moral negotiation dynamic.

In conclusion, this preliminary study contributes to new insights into the understanding of moral judgment in interactive contexts, suggesting that moral negotiation is not simply the sum of individual evaluations; rather, it is a dynamic process involving the integration (and often conflict) of cognitive and emotional processes across both cerebral hemispheres, as well as the neural dynamics of shared morality. This opens up new perspectives for analysing cooperation, disagreement, and the construction of moral meaning in interpersonal relationships.

## Data Availability

The datasets presented in this article are not readily available because of ethical reasons for sensitive personal data protection (requests will be evaluated according to the GDPR - Reg. UE 2016/679 and its ethical guidelines). Requests to access the datasets should be directed to angelica.daffina@unicatt.it.

## References

[ref1] Acconito C. Rovelli K. Saquella F. Balconi M. (2025). Cognitive and emotional engagement in negotiation: insights from EEG and fNIRS Hyperscanning. Exp. Brain Res. 243:151. doi: 10.1007/s00221-025-07093-w, 40397160

[ref2] Angioletti L. Acconito C. Saquella F. Balconi M. (2025). Central (hemodynamic) and peripheral (autonomic) synergy during persuasion within a shared decision-making process. Appl. Sci. 15:1361. doi: 10.3390/app15031361

[ref3] Balconi M. Allegretta R. A. Acconito C. Saquella F. Angioletti L. (2025). The functional signature of decision making across dyads during a persuasive scenario: hemodynamic fNIRS coherence measure. Sensors 25:1880. doi: 10.3390/s25061880, 40293073 PMC11946361

[ref4] Balconi M. Fronda G. (2020). The dialogue between two or more brains: the “Hyperscanning” for organization. Front. Psychol. 11:598332. doi: 10.3389/fpsyg.2020.598332, 33192944 PMC7661773

[ref5] Balconi M. Fronda G. Vanutelli M. E. (2019). Donate or receive? Social hyperscanning application with fNIRS. Curr. Psychol. 38, 991–1002. doi: 10.1007/S12144-019-00247-4

[ref6] Balconi M. Vanutelli M. E. (2017a). Cooperation and competition with Hyperscanning methods: review and future application to emotion domain. Front. Comput. Neurosci. 11:86. doi: 10.3389/fncom.2017.00086, 29033810 PMC5627061

[ref7] Balconi M. Vanutelli M. E. (2017b). Interbrains cooperation: Hyperscanning and self-perception in joint actions. J. Clin. Exp. Neuropsychol. 39, 607–620. doi: 10.1080/13803395.2016.1253666, 27841088

[ref8] Bavelas J. B. Chovil N. Lawrie D. A. Wade A. (1992). Interactive gestures. Discourse Process. 15, 469–489. doi: 10.1080/01638539209544823

[ref9] Cui X. Bray S. Reiss A. L. (2010). Functional near infrared spectroscopy (NIRS) signal improvement based on negative correlation between oxygenated and deoxygenated hemoglobin dynamics. NeuroImage 49, 3039–3046. doi: 10.1016/j.neuroimage.2009.11.050, 19945536 PMC2818571

[ref10] Cui X. Bryant D. M. Reiss A. L. (2012). NIRS-based hyperscanning reveals increased interpersonal coherence in superior frontal cortex during cooperation. NeuroImage 59, 2430–2437. doi: 10.1016/j.neuroimage.2011.09.003, 21933717 PMC3254802

[ref11] Dashtestani H. Zaragoza R. Kermanian R. Knutson K. M. Halem M. Casey A. . (2018). The role of prefrontal cortex in a moral judgment task using functional near-infrared spectroscopy. Brain Behav. 8:1116. doi: 10.1002/brb3.1116, 30253084 PMC6236239

[ref9002] de Gelder B. (2009). Why bodies? Twelve reasons for including bodily expressions in affective neuroscience. Philosophical transactions of the Royal Society of London. Series B, Biological sciences, 364, 3475–3484. doi: 10.1098/rstb.2009.0190 PMC2781896 19884142

[ref12] Decety J. Cowell J. M. (2014). The complex relation between morality and empathy. Trends Cogn. Sci. 18, 337–339. doi: 10.1016/j.tics.2014.04.008, 24972506

[ref13] Enfield N. J. Kita S. de Ruiter J. P. (2007). Primary and secondary pragmatic functions of pointing gestures. J. Pragmat. 39, 1722–1741. doi: 10.1016/j.pragma.2007.03.001

[ref14] Gangemi A. Rizzotto C. Riggio F. Dahò M. Mancini F. (2025). Guilt emotion and decision-making under uncertainty. Front. Psychol. 16:1518752. doi: 10.3389/fpsyg.2025.1518752, 40226489 PMC11985849

[ref15] Giacometti P. Perdue K. L. Diamond S. G. (2014). Algorithm to find high density EEG scalp coordinates and analysis of their correspondence to structural and functional regions of the brain. J. Neurosci. Methods 229, 84–96. doi: 10.1016/J.JNEUMETH.2014.04.020, 24769168 PMC4071772

[ref16] Greene J. D. (2007). Why are VMPFC patients more utilitarian? A dual-process theory of moral judgment explains. Trends Cogn. Sci. 11, 322–323. doi: 10.1016/j.tics.2007.06.004, 17625951

[ref17] Greene J. D. Morelli S. A. Lowenberg K. Nystrom L. E. Cohen J. D. (2008). Cognitive load selectively interferes with utilitarian moral judgment. Cognition 107, 1144–1154. doi: 10.1016/j.cognition.2007.11.004, 18158145 PMC2429958

[ref18] Greene J. D. Nystrom L. E. Engell A. D. Darley J. M. Cohen J. D. (2004). The neural bases of cognitive conflict and control in moral judgment. Neuron 44, 389–400. doi: 10.1016/j.neuron.2004.09.027, 15473975

[ref19] Greene J. D. Sommerville R. B. Nystrom L. E. Darley J. M. Cohen J. D. (2001). An fMRI investigation of emotional engagement in moral judgment. Science 293, 2105–2108. doi: 10.1126/science.1062872, 11557895

[ref20] Haidt J. (2001). The emotional dog and its rational tail: a social intuitionist approach to moral judgment. Psychol. Rev. 108, 814–834. doi: 10.1037/0033-295X.108.4.814, 11699120

[ref21] Hoshi Y. (2016). Hemodynamic signals in fNIRS. Prog. Brain Res. 225, 153–179. doi: 10.1016/bs.pbr.2016.03.004 27130415

[ref22] Hutcherson C. A. Montaser-Kouhsari L. Woodward J. Rangel A. (2015). Emotional and utilitarian appraisals of moral dilemmas are encoded in separate areas and integrated in ventromedial prefrontal cortex. J. Neurosci. 35, 12593–12605. doi: 10.1523/JNEUROSCI.3402-14.2015, 26354924 PMC4563040

[ref23] Kendon A. (2017). Reflections on the “gesture-first” hypothesis of language origins. Psychon. Bull. Rev. 24, 163–170. doi: 10.3758/s13423-016-1117-3, 27439503 PMC5325861

[ref24] Killgore W. D. S. Yurgelun-Todd D. A. (2007). The right-hemisphere and valence hypotheses: could they both be right (and sometimes left)? Soc. Cogn. Affect. Neurosci. 2, 240–250. doi: 10.1093/scan/nsm020, 18985144 PMC2569811

[ref25] Kish-Gephart J. J. Harrison D. A. Treviño L. K. (2010). Bad apples, bad cases, and bad barrels: Meta-analytic evidence about sources of unethical decisions at work. J. Appl. Psychol. 95, 1–31. doi: 10.1037/a0017103, 20085404

[ref26] Koessler L. Maillard L. Benhadid A. Vignal J. P. Felblinger J. Vespignani H. . (2009). Automated cortical projection of EEG sensors: anatomical correlation via the international 10–10 system. NeuroImage 46, 64–72. doi: 10.1016/J.NEUROIMAGE.2009.02.006, 19233295

[ref9001] Kret M. E. De Gelder B. (2012). A review on sex differences in processing emotional signals. Neuropsychologia, 50, 1211–1221. doi: 10.1016/j.neuropsychologia.2011.12.022, 22245006

[ref27] Naseer N. Hong K. S. (2013). Classification of functional near-infrared spectroscopy signals corresponding to the right- and left-wrist motor imagery for development of a brain–computer interface. Neurosci. Lett. 553, 84–89. doi: 10.1016/J.NEULET.2013.08.021, 23973334

[ref28] Naseer N. Hong M. J. Hong K. S. (2014). Online binary decision decoding using functional near-infrared spectroscopy for the development of brain-computer interface. Exp. Brain Res. 232, 555–564. doi: 10.1007/S00221-013-3764-1 24258529

[ref29] Navarick D. J. Moreno K. M. (2022). Moral dilemmas in hospitals: which shooting victim should be saved? Front. Psychol. 13:770020. doi: 10.3389/fpsyg.2022.770020, 35401372 PMC8989733

[ref30] O’Fallon M. J. Butterfield K. D. (2005). A review of the empirical ethical decision-making literature: 1996–2003. J. Bus. Ethics 59, 375–413. doi: 10.1007/s10551-005-2929-7

[ref31] Oostenveld R. Praamstra P. Stegeman D. F. Van Oosterom A. (2001). Overlap of attention and movement-related activity in lateralized event-related brain potentials. Clin. Neurophysiol. 112, 477–484. doi: 10.1016/S1388-2457(01)00460-6, 11222970

[ref32] Palmiotti G. P. Del Popolo Cristaldi F. Cellini N. Lotto L. Sarlo M. (2020). Framing the outcome of moral dilemmas: effects of emotional information. Ethics Behav. 30, 213–229. doi: 10.1080/10508422.2019.1607348

[ref33] Patil I. Zucchelli M. M. Kool W. Campbell S. Fornasier F. Calò M. . (2021). Reasoning supports utilitarian resolutions to moral dilemmas across diverse measures. J. Pers. Soc. Psychol. 120, 443–460. doi: 10.1037/pspp0000281, 31916813

[ref34] Persad G. Wertheimer A. Emanuel E. J. (2009). Principles for allocation of scarce medical interventions. Lancet 373, 423–431. doi: 10.1016/S0140-6736(09)60137-9, 19186274

[ref35] Pinti P. Aichelburg C. Lind F. Power S. Swingler E. Merla A. . (2015). Using Fiberless, wearable fNIRS to monitor brain activity in real-world cognitive tasks. J. Vis. Exp. 2015:53336. doi: 10.3791/53336, 26651025 PMC4692764

[ref36] Quaresima V. Ferrari M. (2019). Functional near-infrared spectroscopy (fNIRS) for assessing cerebral cortex function during human behavior in natural/social situations: a concise review. Organ. Res. Methods 22, 46–68. doi: 10.1177/1094428116658959

[ref37] Reidenbach R. E. Robin D. P. (1988). Some initial steps toward improving the measurement of ethical evaluations of marketing activities. J. Bus. Ethics 7, 871–879. doi: 10.1007/BF00383050

[ref38] Sato H. Yahata N. Funane T. Takizawa R. Katura T. Atsumori H. . (2013). A NIRS–fMRI investigation of prefrontal cortex activity during a working memory task. NeuroImage 83, 158–173. doi: 10.1016/j.neuroimage.2013.06.043, 23792984

[ref39] Sinnott-Armstrong W. (1987). Moral realisms and moral dilemmas. J. Philos. 84:263. doi: 10.2307/2026753

[ref40] Tasso A. Sarlo M. Lotto L. (2017). Emotions associated with counterfactual comparisons drive decision-making in footbridge-type moral dilemmas. Motiv. Emot. 41, 410–418. doi: 10.1007/s11031-017-9607-9

[ref41] Tomasello M. Carpenter M. Call J. Behne T. Moll H. (2005). Understanding and sharing intentions: the origins of cultural cognition. Behav. Brain Sci. 28, 675–691. doi: 10.1017/S0140525X05000129, 16262930

[ref42] Treviño L. K. Weaver G. R. Reynolds S. J. (2006). Behavioral ethics in organizations: a review. J. Manage. 32, 951–990. doi: 10.1177/0149206306294258

[ref43] Tversky A. Kahneman D. (1981). The framing of decisions and the psychology of choice. Science 211, 453–458. doi: 10.1126/science.7455683 7455683

[ref44] Zimeo Morais G. A. Balardin J. B. Sato J. R. (2018). fNIRS optodes’ location decider (fOLD): a toolbox for probe arrangement guided by brain regions-of-interest. Sci. Rep. 2018, 1–11. doi: 10.1038/s41598-018-21716-z, 29463928 PMC5820343

